# White Tea Intake Abrogates Markers of Streptozotocin-Induced Prediabetes Oxidative Stress in Rat Lungs’

**DOI:** 10.3390/molecules26133894

**Published:** 2021-06-25

**Authors:** Ana C. Silveira, Luís Rato, Pedro Fontes Oliveira, Marco G. Alves, Branca M. Silva

**Affiliations:** 1 University of Beira Interior, R. Marquês d’Ávila e Bolama, 6201-001 Covilhã, Portugal; anasilveira1995@gmail.com (A.C.S.); luis.pedro.rato@gmail.com (L.R.); 2 School of Health, Polytechnic Institute of Guarda, 6300-749 Guarda, Portugal; 3 QOPNA & LAQV, Department of Chemistry, University of Aveiro, 3810-193 Aveiro, Portugal; pfobox@gmail.com; 4 Department of Anatomy, Unit for Multidisciplinary Research in Biomedicine (UMIB), Institute of Biomedical Sciences Abel Salazar (ICBAS), University of Porto, Rua de Jorge Viterbo, 4050-313 Porto, Portugal

**Keywords:** white tea, *Camellia sinensis* (L.), antioxidants, oxidative stress, prediabetes, lung

## Abstract

Prediabetes (PrDM) is a prodromal stage of diabetes mellitus (DM) with an increasing prevalence worldwide. During DM progression, individuals gradually develop complications in various organs. However, lungs are suggested to be affected later than other organs, such as the eyes, heart or brain. In this work, we studied the effects of PrDM on male Wistar rats’ lungs and whether the regular consumption of white tea (WTEA) for 2 months contributes to the improvement of the antioxidant profile of this tissue, namely through improved activity of the first line defense antioxidant enzymes, the total antioxidant capacity and the damages caused in proteins, lipids and histone H2A. Our data shows that PrDM induced a decrease in lung superoxide dismutase and glutathione peroxidase activities and histone H2A levels and an increase in protein nitration and lipid peroxidation. Remarkably, the regular WTEA intake improved lung antioxidant enzymes activity and total antioxidant capacity and re-established the values of protein nitration, lipid peroxidation and histone H2A. Overall, this is the first time that lung is reported as a major target for PrDM. Moreover, it is also the first report showing that WTEA possesses relevant chemical properties against PrDM-induced lung dysfunction.

## 1. Introduction

Demographic aging and significant changes in lifestyles, namely in dietary habits, have caused a dramatic increase in Diabetes Mellitus (DM) incidence, representing nowadays one of the greatest threats to public health. In fact, DM is one of the most prevalent metabolic diseases in developed countries. According to recent projections, the prevalence will continue to increase, and it is expected that by 2045 about 629 million adults will suffer from DM [1]. DM generally results from a failure in the secretion and/or action of insulin, a hormone produced by pancreatic β cells that allows glucose to be uptaken by cells, in order to guarantee energy required for metabolism. In prediabetes (PrDM), a prodromal, reversible and less severe condition than DM, the patients present blood glucose levels above the values of healthy people (72–99 mg/dL, but below the values in DM patients (≥126 mg/dL) [1]. In addition to mild hyperglycemia, the patients are glucose intolerant and/or have insulin resistance. This stage of the disease is associated with an increase in oxidative stress (OS) caused by the increment of free radicals production and the reduction of antioxidant defenses, such as superoxide dismutase (SOD), glutathione peroxidase (GPx), glutathione reductase (GR) and catalase (CAT) [2]. High levels of oxidants may alter the function and structure of key biomolecules, such as deoxyribonucleic acid (DNA), proteins, and lipids, leading to cellular injury and death [3]. In DM patients, the lungs seem to be affected later than other organs (like brain, heart and eyes), becoming progressively more susceptible to pneumonia and other respiratory infections, asthma, pulmonary fibrosis, etc. [4]. Ultimately, with all the structural alterations induced by DM, there is a decrease in gas exchange [4]. Thus, it is imperative to find new agents capable of reversing the lungs damage caused by DM or, at least, protecting the lungs from the metabolic dysfunction caused during DM progression. White tea (WTEA), prepared by infusion of *Camellia sinensis* (L.) young leaves/buds, is composed of polyphenols, methylxanthines, organic acids, free amino acids, proteins, polysaccharides, fibers, volatiles, minerals, and many others [5,6,7,8]. From all different types of teas, WTEA is less oxidized since plant materials are steamed and dried immediately after picking [9]. In the production of green tea (GT), the leaves are rolled and steamed to minimize oxidation and inactivate polyphenol oxidase prior to drying [10], while in black tea production the leaves are rolled, disrupting cellular compartmentation, which brings phenolic compounds into contact with polyphenol oxidases, so they undergo oxidation for 90–120 min [9]. This is why WTEA contains relatively high concentrations of catechins and low concentrations of theaflavins and thearubigins [11]. The concentrations of total polyphenols, total catechins, caffeine, gallic acid, theobromine and the members of the flavan-3-ol family, such as (-)-epigallocatechin (EGC), (-)-epicatechin 3-gallate (ECG) and (-)-epigallocatechin 3-gallate EGCG, are significantly higher in the WTEA compared to the other types of tea [11]. The flavan-3-ols (catechins) group, have received much attention among WTEA phytochemicals due to their strong antioxidant, anti-inflammatory, antidiabetic, neuroprotective, anticancer and antimicrobial activities, being considered by many researchers as the main factor responsible for the beneficial health effects of tea and its derivatives [5,6,7,8,12,13,14,15]. WTEA has a great potential as an antidiabetic and antioxidant agent [5,6,7,8,15]. Previous works from our group using a PrDM animal model have also shown the benefits of WTEA consumption in the heart [5], brain [7] and testis [8]. In general, the regular consumption of this tea improved the metabolic profile, increased the total antioxidant capacity and decreased the oxidative damage caused by PrDM in these organs. In the sequence of those reports, the aim of this work was to study the effects of PrDM on the lungs and to evaluate the influence of the regular consumption of WTEA on the activity of first line defense antioxidant enzymes (SOD, GPx, GR, CAT), the total antioxidant capacity and the damages caused in proteins, lipids and histone H2A of PrDM rat lungs.

## 2. Results

### 2.1. Phytochemical Analysis

The most representative class of WTEA phytochemicals was the flavan-3-ol family, with a total content of 330 ± 30 mg/L of WTEA and the most abundant one was EGCG (200 ± 30 mg/L of WTEA) (Table 1). Other compounds were also present in variable amounts: caffeine (180 ± 20 mg/L of WTEA), sucrose (150 ± 10 mg/L of WTEA), EGC (120 ± 10 mg/L of WTEA), L-theanine (48 ± 5 mg/L of WTEA), glucose (15 ± 2 mg/L of WTEA), EC (12 ± 2 mg/L of WTEA), alanine (2 ± 0.2 mg/L of WTEA) and lactate (1 ± 0.1 mg/L of WTEA).

### 2.2. General Characteristics of the Animal Model

Body weight evolution did not show significant variations across groups, since after 60 days of treatment PrDM animals presented 352 ± 32 g and PrDM + WTEA 378 ± 32 g, while the control group showed 347 ± 20 g (see below Table 2). It was observed an increase in food consumption by PrDM animals (26.8 ± 0.39 g), while PrDM + WTEA treated animals consumed 28.6 ± 0.99 g compared to control animals (23.8 ± 0.17 g). Interestingly, PrDM animals ingested more water (35.4 ± 0.22 mL) than control animals (32.0 ± 0.11 mL) (Table 2). Finally, PrDM and PrDM + WTEA animals developed typical type 2 diabetes characteristics. The average glycemia significantly increased from 90 ± 1 mg/dL in rats from the control group to 119 ± 2 mg/dL in PrDM animals and 117 ± 2 mg/dL in PrDM + WTEA, respectively (Table 2). During the treatment no adverse effects were observed in animals, particularly in PrDM group and WTEA treated group.

### 2.3. Glucose Tolerance and Insulin Resistance Tests

**Figure 1:**
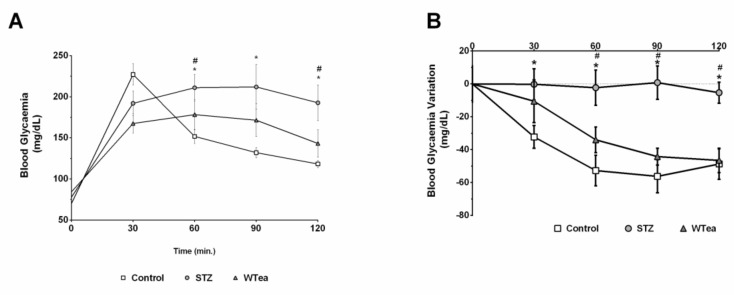
Effect of WTEA consumption by PrDM animals in glucose tolerance and insulin resistance. (**A**) shows blood glucose levels (mg/dL) in control (□), PrDM (◯) and white tea treated animals (▲), measured during the intraperitoneal glucose tolerance test. (**B**) shows blood glucose levels variation (mg/dL) in control (□), PrDM (◯) and white tea treated animals (▲), measured during the intraperitoneal insulin resistance test. Results are expressed as means ± SEM (*n* = 6 for each condition). * Significantly different relatively to control (*p* < 0.05). # Significantly different relatively to PrDM (*p* < 0.05).

The animals were subjected to glucose tolerance and to insulin resistance tests and the area under the curve (AUC) was calculated in order to evaluate those parameters. The animal of the PrDM developed glucose intolerance, since a significantly higher AUC_GTT_ (23364 ± 1095 arbitrary units) was observed, comparatively with the control group (17661 ± 670 arbitrary units) (Table 2, Figure 1A). The insulin resistance test further confirmed that PrDM animals developed typical characteristics of prediabetes, since they presented a significantly lower shift of the blood glycemia when subjected to an insulin resistance test, as observed by AUC_ITT_ (1592 ± 299 arbitrary units) when compared to the control rats (6870 ± 597 arbitrary units) (Table 2, Figure 1B). The PrDM + WTEA animals when subjected to an IP injection of glucose exhibited a significantly smaller AUC_GTT_ value (17760 ± 1446 arbitrary units) than the PrDM group (Table 2, Figure 1A). Furthermore, prediabetic rats drinking white tea also demonstrated a significantly lower insulin resistance (AUC_ITT_ of 4907 ± 871 arbitrary units) compared to water-drinking prediabetic rats (Table 2, Figure 1B). There were no significant differences in the GTT or in the ITT between prediabetic rats that consumed white tea showed, and the control group.

### 2.4. Evaluation of the Effects of WTEA Consumption on the Activity of Lung Antioxidant Enzymes

**Figure 2:**
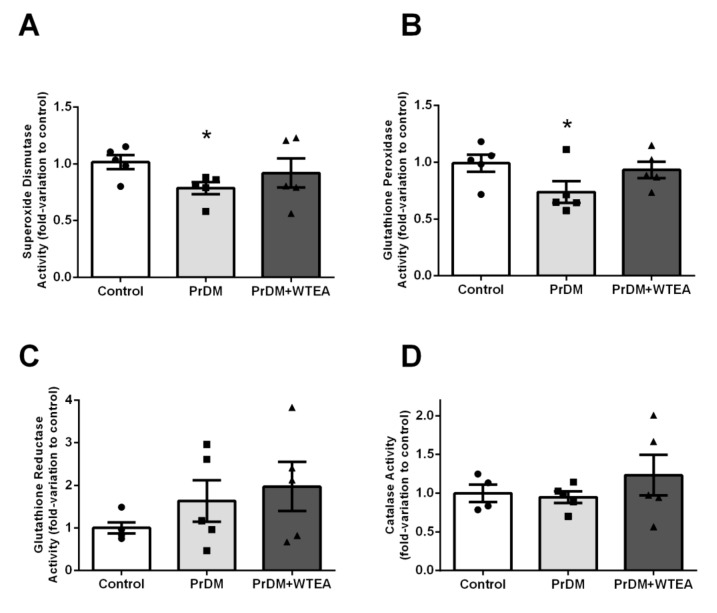
Effect of prediabetes and white tea consumption on lung first line defense antioxidant enzymes activities. (**A**) Superoxide dismutase; (**B**) Gluthatione Peroxidase; (**C**) Gluthatione Reductase; (**D**) Catalase activities in the lung from the control, PrDM and PrDM + WTEA groups. Results are presented with mean ± SEM (*n* = 5 for the control group (●), *n* = 5 for PrDM group (■) and *n* = 5 for WTEA group (▲)). Significant results (*p* < 0.05) relative to the control group are indicated with *.

The results show that PrDM decreased the rats’ lung SOD and GPx activities (0.82 ± 0.06-fold-variation) and 0.65 ± 0.11-fold-variation, respectively). Interestingly, the regular consumption of WTEA for two months led to restoration of SOD and GPx activities to the levels detected in the lung tissue of control group, showing (0.91 ± 0.12-fold-variation and 0.93 ± 0.07-fold-variation to control, respectively) (Figure 2A,B).

No significant alterations were found concerning the activity of GR and CAT enzymes, either in the lung of PrDM rats that consumed water or WTEA (Figure 1C,D)

### 2.5. Evaluation of the Effects of WTEA Consumption on the Antioxidant Parameters

**Figure 3:**
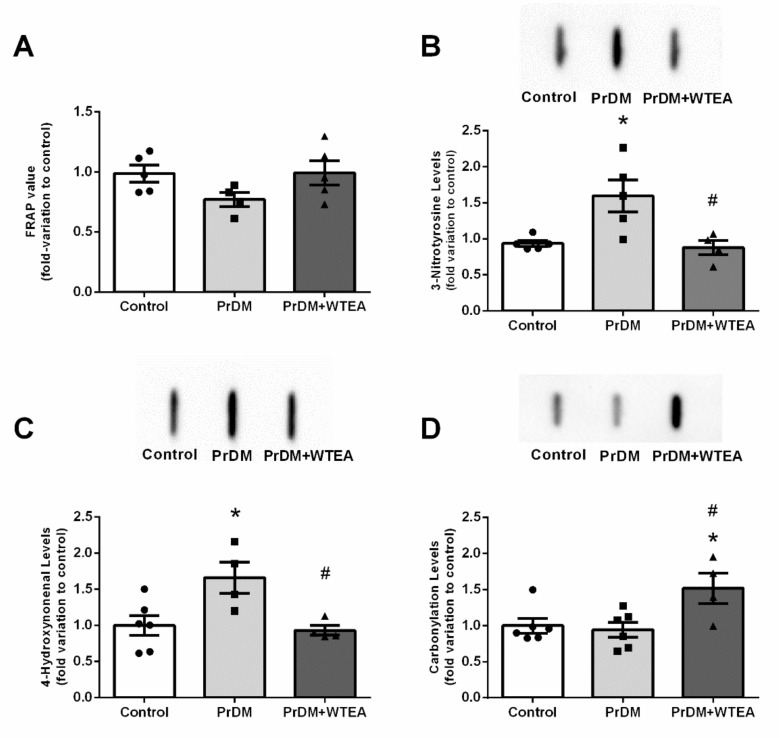
Effect of prediabetes and white tea consumption on lung oxidative stress biomarkers levels. (**A**) Antioxidant capacity, (**B**) protein nitration levels, (**C**) lipid peroxidation levels, and (**D**) carbonyl group levels in lung tissue from the control, PrDM and PrDM + WTEA groups. Results are presented with mean ± SEM (*n* = 6 for the control group (●), *n* = 6 for PrDM group (■) and *n* = 5 for WTEA group (▲)). Significant results (*p* < 0.05) relative to the control group are indicated with * and relative to the PrDM group with #. The insets of panel B, C and D represents an illustrative Slot Blot experiment for the control, prediabetic (PrDM) and prediabetic group drinking White Tea (PrDM + WTEA).

The total antioxidant capacity and the levels of protein carbonyl groups of rat lungs were not significantly affected by PrDM (Figure 3A). Nevertheless, protein nitration significantly increased in PrDM rat lungs (1.60 ± 0.22-fold variation to control) (Figure 3B), as well as lipid peroxidation levels (1.66 ± 0.21-fold variation to control) (Figure 3C).

**Figure 4:**
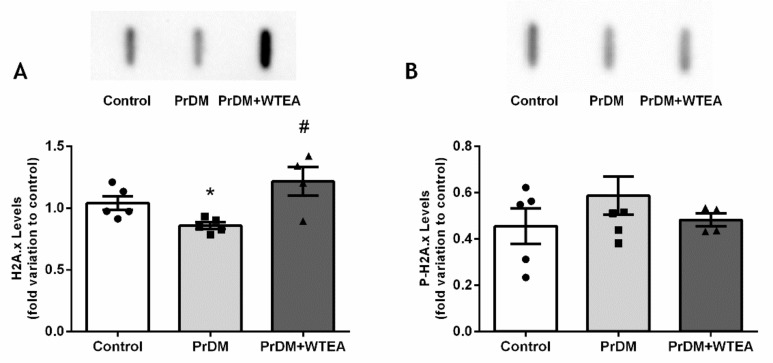
Effect of prediabetes and white tea consumption on lung histone H2A.x and P- H2A.x. (**A**) Histone H2A.x and (**B**) histone P-H2A.x levels in lung tissue from the control, PrDM and PrDM + WTEA groups. Results are presented with mean ± SEM (*n* = 6 for the control group (●), *n* = 6 for PrDM group (■) and *n* = 5 for WTEA group (▲)). Significant results (*p* < 0.05) relative to the control group are indicated with * and relative to the PrDM group with #. The insets of panel A and B represents an illustrative Slot Blot experiment for the control, prediabetic (PrDM) and prediabetic group drinking White Tea (PrDM + WTEA).

In addition, the levels of histone H2A.x in the lung of PrDM rats decreased (0.86 ± 0.03-fold variation to control), while the levels of P-H2A.x did not show significant changes (Figure 4A,B). The regular consumption of WTEA for two months increased the total antioxidant potential of PrDM rats lung tissue from 1.04 ± 0.08 to 1.39 ± 0.11 µmol/mg lung tissue) (Figure 3A). Remarkably, our results also show that WTEA intake significantly decreased lungs protein nitration from 1.60 ± 0.22 to 0.88 ± 0.10-fold variation to control (Figure 3B). In addition, it also reduced lipid peroxidation levels in the lung of PrDM rats drinking WTEA for two months (0.93 ± 0.07-fold variation to control) when compared with those drinking water (1.66 ± 0.21-fold variation to control) (Figure 3C). Yet, the levels of protein carbonyl groups in the lung of PrDM rats drinking WTEA increased (1.52 ± 0.21-fold variation to control) compared to the levels detected in the lung of PrDM rats drinking water (0.95 ± 0.10-fold variation to control) and control rats (1.00 ± 0.10-fold variation) (Figure 3D). In addition, the consumption of WTEA increased the levels of histone H2A.x in the lung (1.22 ± 0.12-fold variation to control) when compared to PrDM rats (0.86 ± 0.03-fold variation to control) (Figure 4A). However, P-H2A.x levels did not show significant changes (Figure 4B).

## 3. Discussion

This study is in line with other works of our research group in different organs of PrDM rats [5,6,7,8]. The animal model currently used was previously developed and characterized by us [5,6,7,8]. Briefly, the rats belonging to the PrDM group developed typical characteristics of PrDM, namely mild hyperglycemia, insulin resistance and glucose intolerance. Although the regular consumption of WTEA had no significant effect on blood glucose levels, it significantly improved glucose tolerance and insulin resistance. This antidiabetic effect of WTEA has been associated with WTEA composition. Our results show that this tea is characterized by the presence of EC, EGC, EGCG, caffeine, L-theanine, alanine, glucose, sucrose and lactate. EGCG and caffeine, the most abundant ones, were probably the main responsible for the glucose intolerance decrease and insulin sensitivity increase [16]. Lung might be a target organ for chronic hyperglycemia once it is highly vascularized and rich in collagen and elastin fibers. We also explored possible deleterious effects of PrDM on the activity of the antioxidant enzymes (SOD, GPx, GR and CAT), the total antioxidant capacity and essential macromolecules of the lung tissue, considering the strong antioxidant and antidiabetic potential of WTEA [17]. SOD converts superoxide radical to hydrogen peroxide (H_2_O_2_) and molecular oxygen (O_2_). H_2_O_2_ is then metabolized by GPx in water (H_2_O) using GR in this process. In addition, CAT also catalyzes the conversion of H_2_O_2_ to H_2_O and O_2_ [2]. Even in a prodromal state of DM, the activities of these endogenous antioxidant enzymes may be reduced. In this study, we found a significant decrease in SOD and GPx activities in the lung tissue of PrDM rats in comparison to the control group. However, no significant alterations were found concerning the activity of GR and CAT. Interestingly, the consumption of WTEA restored the activities of SOD and GPx to levels similar to those detected in the lungs of rats from the control group, possibly due to the presence of phenolic compounds, such as EGCG [18]. Although it is known that this catechin increases the expression of antioxidant enzymes, the underlying mechanism is still unclear. It is thought to be involved in the activation of kinases leading to the phosphorylation of nuclear factor (erythroid-derived 2)-like 2 (NRF2). This transcription factor is then translocated to the nucleus thereby increasing the expression of the antioxidant enzymes [18]. In a previous study, male Balb/c mice treated with a single dose of benzo(a)pyrene (a probable lung cancer-causing agent in humans and animals that induces OS via ROS) that consumed WTEA for 35 days before that treatment presented an increase in SOD, GPx, GR and CAT activities in lung and liver tissues [19]. In our study, WTEA was not as concentrated (once it was prepared using 1 g of WTEA sample per 100 mL of boiling water and not 2 g of WTEA sample per 100 mL of boiling water) and, therefore, provided a lower dose of polyphenols, which may explain the lower effect. After assessing the activities of the antioxidant enzymes, the total antioxidant capacity of the lung tissues was also evaluated. In this study, there was a tendency for the reduction of the lung antioxidant potential in PrDM rats (although not significant), similar to that observed by us in other organs, such as the heart [5], brain [7] and testis [8]. These results suggest that with the reduction of antioxidant defenses, the antioxidant capacity of the lung tissue tends to decrease, favoring an increase in the production of reactive oxygen species (ROS). The consumption of WTEA completely restored the antioxidant potential of the lungs, probably due to the re-established SOD and GPx activities, which may be ascribed to the catechins content of the WTEA [13,20]. Caffeine, an efficient scavenger of hydroxyl radicals, also improves pulmonary function [20]. In this context, ROS can induce the production of carbonyl derivatives and the formation of nitro groups in proteins, as well as the degradation of lipids from the cell membranes (lipid peroxidation). Therefore, protein carbonylation and nitration and lipid peroxidation were used as OS biomarkers. Oxidative alterations of structural proteins and enzymes play a relevant role in the etiology and progression of several chronic diseases, like Alzheimer’s disease, DM, chronic lung disease and bronchopulmonary dysplasia [21]. Our results show that the lungs of PrDM rats presented a significant increase in 3-nitrotyrosine (3-NT) groups. In a condition of PrDM, the free radicals produced in excess react with nitric oxide forming peroxynitrite. Then, this can react with protein tyrosine residues, leading to 3-NT formation [22], which may explain the high levels found in this study. The regular consumption of WTEA decreased the levels of 3-NT in the lung to similar values of the control group. In a study by Pannala et al. [23], EGCG acted as a peroxynitrite scavenger, preventing it from reacting with tyrosine residues and consequently from forming 3-NT, which could also has occurred in our study. Protein carbonyl groups content is one of the most general and well-used biomarkers of severe oxidative protein damage [21]. Our results do not show any significant alterations in the protein carbonyl content in the lungs of PrDM rats. In previous studies we observed a significant increase in protein carbonylation in the heart [5], brain [7] and testis [8] of PrDM rats, that was reverted by the regular intake of WTEA for two months. In an in vitro study by Dias et al. [24], caffeine (5 and 500 µM) had a pro-oxidant effect in human Sertoli cells, leading to an increase in protein carbonylation. Knowing that caffeine is the second most abundant constituent of this tea [25], this methylxanthine may be responsible for the observed results. Concerning the levels of 4-hydroxynonenal (4-HNE), PrDM animals lung showed an increase in lipid peroxidation that was reverted by the regular consumption of WTEA. This is consistent with what was observed by Espinosa et al. [26] where catechins (EC, EGC, EGCG) were able to sequester the peroxyl radical responsible for the formation of 4-HNE. Once WTEA contains these catechins [25], they may be responsible for this protective effect. Histones are proteins that package DNA into nucleosomes and histone H2A is one of the main ones involved in the structure of eukaryotic cells chromatin. In fact, histone H2A is important for packaging DNA into chromatin, once it affects gene expression, and is involved in the mechanisms of DNA repair, which means that decreased histone H2A levels are associated with DNA damage [27]. In this study, we found that the levels of histone H2A were decreased in the lung of rats from the PrDM group. Interestingly, WTEA consumption restored histone H2A levels demonstrating the protecting activity of this tea against PrDM-induced DNA damage on lung. Accordingly, studies by Kumar et al. [19] suggest that WTEA attenuates OS and DNA damage caused by benzo(a)pyrene by increasing the antioxidant defenses of pulmonary tissue.

## 4. Materials and Methods

### 4.1. Chemicals

The chemicals were purchased from Sigma-Aldrich (St. Louis, MO, USA) except those specifically indicated below. Anti-3-nitrotyrosine antibody (9691S), H2A.X Rabbit (2595S) and Phospho-Histone H2A.X Rabbit (9718S) were purchase from Cell Signalling (Danvers, MA, USA). The anti-4-hydroxynonenal (AB5605) antibody was purchased from Millipore Corporation (Billerica, MA, USA). Goat anti-rabbit IgG-HRP (A2315) and mouse anti-goat IgG-HRP (Sc-2354) were purchased from Santa Cruz Biotechnology (Dallas, TX, USA). The Western Bright ECL substrate was purchased from Advansta (Menlo Park, CA, USA).

### 4.2. Preparation of White Tea and Determination of White Tea Composition

WTEA from a commercial brand was prepared daily, according to the manufacturer’s instructions. Briefly, samples were subjected to infusion (1 g/100 mL of distilled boiling water) during 3 min. The infusion was filtered through a 0.2 μm sterile syringe filter of cellulose acetate (VWR, PA, USA). For determination of WTEA composition, 5 different extracts were analyzed by Proton Nuclear Magnetic Resonance (^1^H-NMR) as previously described [5,6,7,8]. In brief, ^1^H-NMR spectra of WTEA samples were acquired at 14.1 T, 25 °C, using a Bruker Avance 600 MHz spectrometer (Bruker BioSpin, Rheinstetten, Germany). Sodium fumarate (final concentration of 1 mM) was used as an internal reference (6.50 ppm) to quantify the following phytochemicals (multiplet, ppm): L-theanine (triplet, 1.08); lactate (doublet, 1.33); alanine (doublet, 1.45); (-)-epigallocatechin-3-gallate (EGCG) (doublet, 2.7); caffeine (singlet, 3.29); H1-α-glucose (doublet, 5.22); sucrose (doublet, 5.4); (-)-epigallocatechin (EGC) (singlet, 6.6); (-)-epicatechin (EC) (singlet, 7.0). The relative areas of 1 H NMR resonances were quantified using the curve-fitting routine supplied with the NUTSproTM NMR spectral analysis program (Acorn, NMR Inc., Fremont, CA, USA).

### 4.3. Animal Model and Experimental Design

In this study, eighteen male Wistar rats were obtained from a colony of accredited animals (Health Sciences Research Centre, University of Beira interior). All animal experiments were performed according to the "Guide for the Care and Use of Laboratory Animals" published by the US National Institutes of Health (NIH Publication No. 85-23, revised 1996) and the rules for the care and handling of laboratory animals (Directive 2010/63/UE). In brief, 2-days-old male Wistar rats from the PrDM group were injected with streptozotocin (STZ) (40 mg/kg, IP) freshly diluted in citrate buffer (0.1 M-sodium citrate, pH 4·5). The control group received only the vehicle solution in an equivalent volume (*n* = 6). Rats were fed ad libitum with a standard chow diet (4RF21 certificate; Mucedola). At 1 month of age, STZ-treated rats were randomly divided into two groups, and one group consumed WTEA ad libitum for 2 months (PrDM + WTEA group; *n* = 6). The other group of STZ-treated rats (PrDM group; *n* = 6) and control rats (control group; *n* = 6) were also randomly divided and consumed water for 2 months. AT the end of the experience (at three months of age), the rats were submitted to a glucose tolerance test (GTT) and an insulin tolerance test (ITT) according to [5,7], Oliveira et al. [8] and Dias et al. [6] (for more details see Figure 1). At the end of the treatment, rats were killed by decapitation. The lungs were removed, weighed, snap frozen and stored at –80 °C.

### 4.4. Enzymatic Assays

#### 4.4.1. Superoxide Dismutase Activity

Briefly, at 25 °C, 50 μg of protein of the lung tissue (in 216 mM phosphate buffer) were added to the reaction cocktail (pH 7.8 containing distilled H_2_O, 216 mM phosphate buffer, 10.7 mM ethylenediaminetetraacetic acid (EDTA) solution, cytochrome C solution 1.1 mM and 0.108 mM xanthine solution). The reaction was started by adding 0.05 units/mL xanthine oxidase enzyme solution and increasing the absorbance at 550 nm for 5 min in the xMark Microplate Absorbance Spectrophotometer from Bio-Rad (Hercules, CA, USA). The enzyme activity in units/mg of protein was calculated according to the experimental protocol used. Absolute values of the Superoxide Dismutase activity assay can be found in Table S1 of the Supplementary Materials.

#### 4.4.2. Gluthatione Peroxidase Activity

Briefly, at 25 °C, GPx assay buffer (50 mM Tris HCl, pH 8.0 containing 0.5 mM EDTA) was placed in a cuvette. Subsequently, 0.25 mM of Nicotinamide adenine dinucleotide phosphate (NADPH) assay reagent (5 mM NADPH, 42 mM Reduced glutathione, and 10 units/mL of GR) and 50 μg of protein of the lung tissue sample were added to the previously diluted in GPx assay buffer. The reaction was started with the addition of 300 µM tert-Butyl hydroperoxide solution and the decrease in absorbance at 340 nm measured on Ultrospec™ 3000, Pharmacia Biotech (Cambridge, England) during 1 min. The enzyme activity in units/mg of protein was calculated according to the experimental protocol. Absolute values of the Gluthatione Peroxidase activity assay can be found in Table S2 of the Supplementary Materials.

#### 4.4.3. Gluthatione Reductase Activity

Briefly, at 25 °C, 2 mM GSSG and GR assay buffer was placed in a 96-well plate. Then, 50 μg of protein of the lung tissue was added in GR dilution buffer and 3 mM 5.5’-dithiobis (2-nitrobenzoic acid). The reaction was started by adding 2 mM NADPH and increasing the absorbance at 412 nm for 1 min and 30 s on the xMark Microplate Absorbance Spectrophotometer from Bio-Rad (Hercules, CA, USA). The enzyme activity in units/mg of protein was calculated according to the experimental protocol. Gluthatione Reductase activity assay can be found in Table S3 of the Supplementary Materials.

#### 4.4.4. Catalase Activity

A standard curve was prepared through a series of standard solutions of hydrogen peroxide (H_2_O_2_) whose concentrations were 0, 0.0125, 0.0250, 0.0500, 0.0750 mM in the reaction mixture diluted in Assay Buffer (50 mM potassium phosphate buffer, pH 7.0). The above solutions were added to a 96-well plate together with reagent color (150 mM potassium phosphate buffer, pH 7.0, containing 0.25 mM 4-aminoantipyrine and 2 mM 3,5-dichloro-2-hydroxybenzenesulfonic acid). After 15 min, the absorbance at 520 nm was read in the xMark Microplate Absorbance Spectrophotometer from Bio-Rad (Hercules, CA, USA). For the samples, 0.3 μg of protein of lung tissue was prepared in assay buffer and placed in the wells. The reaction was started by the addition of Colorimetric Assay Substrate Solution 200 mM H_2_O_2_ and incubated for 3 min. After that time the reaction was stopped by Stop Solution (sodium azide 15 mM) and transferred to a new well. Reagent color was added and remained for 15 min at room temperature. Absorbances were measured from the same method used for standard curve solutions. The enzyme activity in mmol/min/mL of protein was calculated according to the experimental protocol used. Absolute values of the Catalase activity can be found in Table S4 of the Supplementary Materials.

### 4.5. The Antioxidant Activity By FRAP Assay

The ferric reducing antioxidant power (FRAP) was performed according to the colorimetric method described by Benzie and Strain [13] for each of the lung samples. Briefly, the tissue samples were homogenized in lysis buffer. Protein quantification was determined by the Bradford microassay. The FRAP reaction medium was prepared from a mixture of acetate buffer (300 mM, pH 3.6), 2,4,6-Tripyridyl-S-Triazine (TPTZ) (10 mM in 40 mM HCl) and iron trichloride (20 mM) in a ratio of 10:1:1 (*v*/*v*/*v*). The Fe^3+^—TPTZ complex was reduced to Fe^2+^—TPTZ and measured the absorbance at 595 nm in the xMark Microplate Absorbance Spectrophotometer from Bio-Rad (Hercules, CA, USA) 40 min after sample addition. Absolute values of the FRAP assay can be found in Table S5 of the Supplementary Materials.

#### 4.5.1. Analysis of Oxidative Stress Biomarkers

To evaluate the oxidative parameters, the analysis of the protein nitration groups, lipid peroxidation and histone H2A levels was used. For this, a Slot blot technique was performed. A total of 5 µg of lung protein were diluted in PBS and transferred to PVDF membranes using a Hybri-Slot Manifold System (Biometra, Göttingen, Germany). The membranes were then incubated overnight at 4 °C with nitro-tyrosine antibody (1:5000, 9691S), anti-4-hydroxynonenal antibody (1:5000, AB5605), H2A.X Rabbit (1:1000, 2595S) and Phospho-Histone H2A.X Rabbit (1:1000,9718S). For the protein nitration, H2A levels and P-H2A levels the samples were visualized using goat anti-rabbit IgG-HRP (1:10000, A2315), for the lipid peroxidation groups, the mouse anti-goat IgG-HRP (1:5000, Sc-2354) was used. The membranes were reacted with WesternBright™ ECL and visualized on the Chemidoc MP Imaging System from Bio-Rad (Hercules, CA, USA). Densities of each band were obtained with Image Lab Software 5.1 from Bio-Rad (Hercules, CA, USA).

The carbonyl groups were determined as described by Rato et al. [28]. To perform this analysis a Slot blot assay was also performed. Membranes were then incubated overnight at 4 °C with rabbit anti-DNP antibody (1:5000, D9656). Samples were visualized through the use of rabbit IgG-HRP (1:10000, A2315). The membranes were reacted and visualized as described in [28]. Absolute values of the oxidative stress biomarkers assay can be found in Table S6 of the Supplementary Materials.

#### 4.5.2. Statistical Analysis

Statistical significance was assessed by one-way ANOVA, followed by Bonferroni post-test using GraphPad Prism 5 (GraphPad Software, San Diego, CA, USA). All data are presented as mean ± SEM. Differences with *p* < 0.05 were considered statistically significant.

## 5. Conclusions

As far as we know, this is the first study demonstrating that lung is a target organ for PrDM, once its oxidative status is adversely affected by mild hyperglycemia, insulin resistance and glucose intolerance. Remarkably, the regular consumption of WTEA for two months reversed the oxidative deleterious damages promoted by PrDM on the pulmonary tissue, namely by re-establishing the SOD and GPx activities and the levels of protein nitration, lipid peroxidation and histone H2A. Yet, the increase in lung protein carbonylation inflicted by WTEA intake shows that more research needs to be performed before WTEA can be suggested as a cost-effective and safe antioxidant therapy for those who developed DM or are at risk for developing this disease.

## Figures and Tables

**Table 1:** White tea composition determined by ^1^H-NMR.

Compound	Content (mg/L)
**EGCG**	200 ± 30
Caffeine	180 ± 20
Sucrose	150 ± 10
**EGC**	120 ± 10
L-theanine	48 ± 5
Glucose	15 ± 2
**EC**	12 ± 2
Alanine	2 ± 0.2
Lactate	1 ± 0.1

Legend: EGCG—(-)-epigallocatechin-3-gallate; EGC—(-)-epigallocatechin; EC—(-)-epicatechin. Members of the flavan-3-ol family at bold. Results are presented as mean ± S.E.M. (*n* = 5).

**Table 2:** Average values of weight, blood glycemia, water/white tea intake and food consumption/animal/day, area under the curve for glucose tolerance (AUC_GTT_) and insulin tolerance (AUC_ITT_) tests in rats from the control, PrDM and PrDM + WTEA after 60 days of treatment.

Parameters	Control Group	PrDM Group	PrDM + WTEA Group
Weight (g)	347 ± 20	352 ± 32	378 ±32
Glycemia (mg/dL)	90 ± 1	119 ± 2 ^*^	117 ± 2 ^*^
Food consumption (g)	23.8 ± 0.17	26.8 ± 0.39 ^*^	28.6 ± 0.9 ^*#^
Drink Intake (mL)	32.0 ± 0.11	35.4 ± 0.22 ^*^	31.0 ± 0.35 ^#^
AUC_GTT_	17661 ± 670	23364 ± 1095 ^*^	17760 ± 1446 ^#^
AUC_ITT_	6870 ± 597	1592 ± 299 ^*^	4907 ± 871 ^#^

Legend: PrDM—prediabetic; PrDM + WTEA—prediabetic treated with white tea. Results are expressed as means ± SEM (*n* = 6 for each condition). * Significantly different relatively to control (*p* < 0.05). ^#^ Significantly different relatively to PrDM (*p* < 0.05).

## Data Availability

Not applicable.

## Supplementary Materials

The following are available online, Table S1: Absolute values of Superoxide Dismutase activity in the lung from the Control, PrDM and PrDM + WTEA groups, Table S2: Absolute values of Glutathione Peroxidase activity in the lung from the Control, PrDM and PrDM + WTEA groups, Table S3: Absolute values of Glutathione Reductase activity in the lung from the Control, PrDM and PrDM + WTEA groups, Table S4: Absolute values of Catalase activity in the lung from the Control, PrDM and PrDM + WTEA groups, Table S5: Absolute values of FRAP assay in the lung from the Control, PrDM and PrDM + WTEA groups, Table S6: Absolute values of Nitration, Peroxidation, Carbonylation, H2A.x and P-H2A.x of Slot Blot experiment from the lung tissue of the Control, PrDM and PrDM + WTEA groups.
