# Supplementary material for: White Tea Intake Abrogates Markers of Streptozotocin-Induced Prediabetes Oxidative Stress in Rat Lungs’
Source: Molecules. 2021 Jun 25;26(13):3894. doi: 10.3390/molecules26133894 (PMC8271685; doi:10.3390/molecules26133894)
Supplement: Supplementary file 1 [file molecules-26-03894-s001.zip › molecules-1240212-supplementary.pdf]

Supplementary material

Table S1: Absolute values of Superoxide Dismutase activity in the lung from the Control, PrDM and PrDM + WTEA groups. Values are in Units/mg of protein.

Groups	Samples	Values
CTR	1	1777,77
	2	1449,27
	3	1666,66
	4	2083,33
	5	2000,00
	6	-
PrDM	1	1466,66
	2	1052,63
	3	1555,55
	4	1594,20
	5	1871,34
	6	-
PrDM+WTEA	1	1458,33
	2	1018,51
	3	2222,22
	4	1434,78
	5	2183,23
	6	-

Table S2: Absolute values of Glutathione Peroxidase activity in the lung from the Control, PrDM and PrDM + WTEA groups. Values are in Units/mg of protein.

Groups	Samples	Values
CTR	1	1,57
	2	0,77
	3	1,53
	4	0,26
	5	1,27
	6	-
PrDM	1	0,25
	2	0,69
	3	1,20
	4	0,77
	5	0,70
	6	-
PrDM+WTEA	1	1,11
	2	0,79
	3	0,96
	4	0,94

	5	1,24
	6	-

Table S3: Absolute values of Glutathione Reductase activity in the lung from the Control, PrDM and PrDM + WTEA groups. Values are in Units/mg of protein.

Groups	Samples	Values
CTR	1	0,08
	2	0,04
	3	0,05
	4	0,04
	5	0,05
	6	-
PrDM	1	0,06
	2	0,02
	3	0,15
	4	0,14
	5	0,12
	6	-
PrDM+WTEA	1	0,03
	2	0,04
	3	0,12
	4	0,10
	5	0,18
	6	-

Table S4: Absolute values of Catalase activity in the lung from the Control, PrDM and PrDM + WTEA groups. Values are in µmol/min/mL.

Groups	Samples	Values
CTR	1	0,08
	2	0,04
	3	0,05
	4	0,04
	5	0,05
	6	-
PrDM	1	0,06
	2	0,02
	3	0,15
	4	0,14
	5	0,12
	6	-
PrDM+WTEA	1	0,03
	2	0,04
	3	0,12
	4	0,10

	5	0,18
	6	-

Table S5: Absolute values of FRAP assay in the lung from the Control, PrDM and PrDM + WTEA groups. Values are in µmol of antioxidant activity/ mg of lung tissue.

Groups	Samples	Values
CTR	1	1,85
	2	1,50
	3	1,12
	4	1,13
	5	1,31
	6	-
PrDM	1	1,01
	2	0,82
	3	1,20
	4	0,00
	5	1,83
	6	-
PrDM+WTEA	1	1,28
	2	1,61
	3	0,71
	4	1,15
	5	1,52
	6	-

Table S6: Absolute values of Nitration, Peroxidation, Carbonylation, H2A.x and P-H2A.x of Slot Blot experiment from the lung tissue of the Control, PrDM and PrDM + WTEA groups. Values correspond to the optical density of the bands.

Groups	Samples	Nitration Values	Peroxidation Values	Carbonylation Values	H2A.x Values	P-H2A.x Values
CTR	1	1285990	1285990	755502	2748176	2748176
	2	690456	690456	503946	749596	749596
	3	1392402	1392402	809376	828240	828240
	4	1254454	1254454	741836	415296	415296
	5	2257562	2257562	338990	2503716	2503716
	6	986084	986084	688714	730032	730032
PrDM	1	699964	699964	282322	1144512	1144512
	2	422216	422216	838112	507088	507088
	3	1984572	1984572	888948	131644	131644
	4	1952950	1952950	893226	685012	685012
	5	1932228	1932228	545944	1478008	1478008
	6	1208372	1208372	1121262	680140	680140
PrDM+WTEA	1	1036712	1036712	1643654	701404	701404
	2	1165786	1165786	1984032	574832	574832

	3	1393932	1393932	1374276	580848	580848
	4	1521186	1521186	2392982	1079796	1079796
	5	1506950	1506950	1298696	710148	710148
	6	-	-	-	-	-
